# Hyphenated Mass Spectrometry versus Real-Time Mass Spectrometry Techniques for the Detection of Volatile Compounds from the Human Body

**DOI:** 10.3390/molecules26237185

**Published:** 2021-11-26

**Authors:** Oliver Gould, Natalia Drabińska, Norman Ratcliffe, Ben de Lacy Costello

**Affiliations:** 1 Centre for Research in Biosciences, Frenchay Campus, University of the West of England, Coldharbour Lane, Bristol BS16 1QY, UK; norman.ratcliffe@uwe.ac.uk (N.R.); ben.delacycostello@uwe.ac.uk (B.d.L.C.); 2 Department of Chemistry and Biodynamics of Food, Institute of Animal Reproduction and Food Research, Polish Academy of Sciences, 10-748 Olsztyn, Poland; 3 Food Volatilomics and Sensomics Group, Faculty of Food Science and Nutrition, Poznan University of Life Sciences, 60-637 Poznan, Poland

**Keywords:** mass spectrometry, chromatography, SIFT-MS, SESI-MS, PTR-MS, GC-MS

## Abstract

Mass spectrometry (MS) is an analytical technique that can be used for various applications in a number of scientific areas including environmental, security, forensic science, space exploration, agri-food, and numerous others. MS is also continuing to offer new insights into the proteomic and metabolomic fields. MS techniques are frequently used for the analysis of volatile compounds (VCs). The detection of VCs from human samples has the potential to aid in the diagnosis of diseases, in monitoring drug metabolites, and in providing insight into metabolic processes. The broad usage of MS has resulted in numerous variations of the technique being developed over the years, which can be divided into hyphenated and real-time MS techniques. Hyphenated chromatographic techniques coupled with MS offer unparalleled qualitative analysis and high accuracy and sensitivity, even when analysing complex matrices (breath, urine, stool, etc.). However, these benefits are traded for a significantly longer analysis time and a greater need for sample preparation and method development. On the other hand, real-time MS techniques offer highly sensitive quantitative data. Additionally, real-time techniques can provide results in a matter of minutes or even seconds, without altering the sample in any way. However, real-time MS can only offer tentative qualitative data and suffers from molecular weight overlap in complex matrices. This review compares hyphenated and real-time MS methods and provides examples of applications for each technique for the detection of VCs from humans.

## 1. Introduction

Mass spectrometry (MS) has been used for many applications [1], including environmental [2], biomedical [3,4], security [5], forensic science [6], space exploration [7], and agri-food [8,9] applications, among numerous others, over the years since its inception at the beginning of the 20th century. There are multiple applications for MS within each field, including proteomics [10], lipidomics [8], and metabolomics [11]. MS is used in connection with many instruments, such as gas chromatography (GC), liquid chromatography (LC), and matrix-assisted laser desorption/ionisation (MALDI). Each has its advantages and limitations and are better suited for specific applications over others.

Volatile compounds (VCs) are a heterogeneous group of compounds that demonstrate a high vapour pressure at room temperature. VCs are emitted by the cells of the human body as products of their metabolism and changes during metabolic disturbances in the body. Therefore, the measurement of VCs in biological fluids as a potential diagnostic tool has gained increased amounts of attention in the last few years. 

Volatilomics consists of the analysis of the profile of the volatile and semi-volatile compounds in biological fluids [12]. This is potentially an innovative tool for the non-invasive detection and monitoring of diseases and for evaluating the effectiveness of their treatment. VCs can be detected in all body fluids, and their abundance differs significantly depending on the biological fluid [13]. The majority of the VCs detected in the human body are endogenous molecules (e.g., isoprene); however, some can come from both endogenous and exogenous sources (e.g., acetone) or may mainly be exogenous (e.g., toluene, acetonitrile). The VCs detected in the human body comprise a wide range of chemical classes, including aldehydes, ketones, alcohols, hydrocarbons, sulphur- and nitrogen-nitrogen containing compounds, and many others [13]. 

Shirasu and Touhara [14] briefly outline the basis for the use of VCs in breath, skin and sweat, urine, and faeces. In short, breath VCs are derived from the ingestion of food, beverages, cigarettes, and gases in the atmosphere and exhaled blood-borne compounds. Skin VCs are mostly derived from sweat, and in this case, most of the VCs derived from the internal metabolism are altered by the bacteria on the skin. Urinary VCs are primarily derived from metabolic processes throughout the body and contain various functional groups such as ketones, alcohols, and furans; importantly, it is possible for urinary VCs to be affected by ingested food and beverages. The stool comprises bacteria that generate a significant number of VCs, meaning that stool sample analysis has excellent potential for the diagnosis of gastrointestinal disorders [14]. 

Amann et al. [15] compiled a list of all of the published VCs found in the breath, skin emanations, urine, faeces, and saliva from healthy humans. This paper states that in the years leading up to 2014, 874 compounds were reported in the breath, 279 compounds were determined from urine, 504 were determined from skin emanations, 353 were determined from saliva, 130 compounds were determined from blood, and 381 compounds were determined from faeces. The recent update [13] of the VCs comprising the human volatilome showed that this number had increased by 900. The new total VCs reported for each bodily fluid are 379 (blood), 1488 (breath), 443 (faeces), 290 (milk), 549 (saliva), 196 (semen), 623 (skin), and 444 (urine). Many publications link VCs to certain disease states, and the majority of this work is undertaken using MS-based techniques.

Understanding the pathways by which VCs are generated in the body is the key to understanding the mechanisms by which VCs can have clinical utility. Miekisch et al. [16] described the production mechanism for many families of compounds found in the breath. For instance, acetone is a very commonly occurring compound in breath and is generally accepted to be derived from the decarboxylation of acetyl-CoA by the liver. Elevated breath acetone is considered to be an indicator of beta-hydroxybutyrate in the blood [17] and has been attributed to type 1 and type 2 diabetes in a number of studies [18,19,20]. However, despite the biochemical mechanisms for breath acetone being understood, there are limits to its clinical utility, primarily because the acetone level in the breath of healthy participants is very variable [20]. Many of the possible metabolic routes for the production of certain VCs derived from the oxidation of fatty acids were explained in a recent study by our group [21].

The most selected analytical method for identifying VCs from humans is GC-MS, and it has been some years since the various methodologies have been compared for this application. Several sample preparation methods were described, with solid-phase microextraction (SPME) being the most frequently used [22]. Other sample preparation techniques used for VCs analysis include thermal desorption (TD), headspace (HS), stir bar sorptive extraction (SBSE) and solid-phase dynamic extraction (SPDE), single-drop microextraction (SDME), and liquid phase microextraction (LPME). The new field of vacuum-assisted sampling techniques, such as vacuum-assisted sorbent extraction (VASE), and the modification of the above-mentioned techniques under reduced pressure, has gained more attention in recent years [23,24]. The characteristics and comparison of the various extraction methods have been repeatedly reviewed [25,26,27]; thus, these will not be described in this review in detail.

In many cases, VC analysis can be improved using real-time online techniques such as selected ion flow tube-mass spectrometry (SIFT-MS) and proton transfer reaction mass spectrometry (PTR-MS), both of which overcome the need for sample pre-concentration, which can introduce contaminants or can degrade the sample [28]. Ratiu et al. [29] present a review of mass spectrometry techniques for the VC analysis of compounds generated by bacteria. This review details many of the general advantages and disadvantages that are associated with the various techniques and compares the costs of the different instruments. However, there is no comprehensive review on the comparison of the hyphenated and real-time MS techniques for VC analysis for diagnostics. Therefore, this review addresses this issue and outlines the principles and mechanisms of the various MS techniques and their biomedical applications.

## 2. Hyphenated Mass Spectrometry Techniques

The chromatography phase of these techniques allows for the separation of compounds, making the identification and quantification of compounds possible. There are important decisions to be made about sample preparation and pre-concentration to ensure the most efficient VC extraction without introducing confounding variables. In general, the chromatographic separation phase can have a relatively long per sample analysis time compared to real-time techniques; however, some improvements have been proposed.

GC is the most frequently used separation technique to analyse VCs. The comparison of different modifications of MS used for VCs analysis is presented in Table 1. Electron impact ionisation (EI) is considered to be an example of hard ionization and is most commonly used for chromatographic MS methods.

### 2.1. Gas Chromatography-Mass Spectrometry (GC-MS)

GC-MS with a single quadrupole is still largely considered to be the gold standard for qualitative gas analysis due to the comprehensive mass spectral libraries and deconvolution software available as well as the high availability of the instrument. There is such a vast quantity of biomedical-based GC-MS papers that it is difficult to overestimate the role of GC-MS in advancing volatilomics.

In GC-MS, the compounds elute from the chromatographic column at retention times that are related to their molecular mass and chemical properties, such as polarity. These compounds are ionised as they enter the mass analyser, allowing a spectrum to be mapped based on the mass to charge ratio (*m*/*z*) of the product ions. This ionisation process makes this technique ideal for identifying the compounds that are present in complex matrices. 

The National Institute of Standards and Technology (NIST) library software is able to identify the compounds that are present by comparing the spectra that are produced from the ionised products of the analyte with those in the library. However, the match made by the NIST library can be incorrect, and the library only provides tentative peak assignment. Therefore, in the majority of cases, standards or high-resolution accurate-mass instruments are required to confirm the identification with greater confidence. 

Most modern GC-MS instruments have reported limits of detection (LOD) at the femtogram level. However, when running a typical sample using a general method in total ion count mode, most users report LODs at the picogram level [30]. While GC-MS provides good sensitivity and is the most commonly used qualitative method for trace gas detection, quantification requires considerable method development and standard analysis.

There have been numerous publications over many years that have attempted to utilise GC-MS for diagnostic purposes. For instance, Tait et al. [31] used SPME GC-MS to identify *Clostridium difficile* from the headspace of stool samples and achieved a reported sensitivity and specificity of 83.1% and 100%, respectively. This study identified 2-fluoro-4-methylphenol, isobutyric acid, butyric acid, isocaproic acid, and p-cresol as the key compounds [31]. Another group also used SPME GC-MS to differentiate the causes of diarrhoea and found that 5-methyl-2-furancarboxaldehyde was associated with *Clostridium difficile* [32]. This association improved when coupled with the absence of 3-methylindole [32]. However, the study was performed with only 35 participants, of which only 6 were confirmed *Clostridium difficile* patients. Therefore, the results require confirmation.

**Table 1:** The advantages and limitations of the GC-based hyphenated MS techniques.

Instrument	Advantages	Limitations
GC-MS	Capable of providing detailed data from complex matrices. The most cost-effective method of hyphenated GC-based MS analysis. Can tailor pre-concentration methods to suit the sample being analysed. Generally easy to operate once set up and data easy to analyse. Good reproducibility. Miniaturisation efforts were achieved.	Samples require pre-processing and pre-concentration, which can be time consuming and expensive. Sample analysis can typically range from 30–60 min and requires blank runs and QC qualification, limiting throughput. The lowest sensitivity among MS techniques used with GC. Commonly, problems with identification if run without standards. Standards required for quantification.
GC-MS/MS	Much greater selectivity and sensitivity versus GC-MS. Can measure a wide mass range, including peptides. Enhanced signal-to noise ratio. Can provide more accurate identification of compounds versus GC-MS.	Both expensive and has a large lab. footprint. Requires experienced personnel for both method development and data analysis. Low mass resolution.
GC-TOFMS	Great mass resolution allows for more accurate identification of compounds. Has a theoretically unlimited upper mass range. Very high sensitivity. More consistent performance for low concentration compounds. Rapid acquisition rates.	At high signal-to-noise levels, reproducibility suffers. Poor detector linearity compared to a standard quadrupole MS. Limited dynamic range.
GC × GC-TOFMS	Retains the same advantages as GC-TOFMS in addition to the following: Compatible with single GC injection techniques. Can provide sharper and better resolved peaks. Solves some issues of co-elution compared to GC-MS. Higher and more effective use of peak capacity when compared to one-dimensional GC methods and results in improved signal-to-noise ratios due to increased signal (focusing/band compression at modulator). Decreased noise (separation of analytes from primary column bleed and coeluting analytes). Therefore, spectra are cleaner, allowing improved compound identification. The combination of GC × GC with a TOFMS is considered the most powerful tool for qualitative VC analysis.	Retains the same disadvantages of GC-TOFMS in addition to the following: Long sample run times compared to techniques with single chromatographic separation. If the modulation between the two columns is not properly set, then separation is compromised. Much higher per sample costs. Higher cost of the instrument.

GC-MS was applied to detect VC profiles linked to cancer diagnosis. Altomare et al. [33] used TD GC-MS to analyse the breath from 78 participants (37 cancer patients) in an attempt to differentiate colorectal cancer from healthy controls. The VC patterns were introduced into the probabilistic neural network, resulting in an overall accuracy of 85%. Although the accuracy dropped during a validation phase to 76%, this is still an encouraging result. In another study, Amal et al. [34] also analysed the VCs in breath and demonstrated that acetone and ethyl acetate were increased in cancer patients versus controls and that ethanol and 4-methyl octane were decreased in patients with cancer.

More recently, Phillips et al. [35] analysed the breath from 178 women that had undergone screening for breast cancer; a total of 54 represented cancer cases, and 124 were controls. The GC-MS analysis yielded a test accuracy of 90% (77% cross validated). The group compared these findings with a GC surface acoustic wave detector, which produced a lower overall accuracy of 86% (74% cross-validated). Importantly searching the literature revealed very few studies that included more than 100 samples, the absence of large-scale studies is a significant factor when attempting to comment on the clinical utility of volatile diagnostics.

In another study, a (liquid–liquid extraction) LLE method was optimised for GC-MS analysis, which showed that LLE was capable of the detection of a broader range of compounds and that it is able to overcome some of the selectivity issues that exist with SPME [36]. The optimised protocol proposed by the authors allowed for the detection of more than 330 VCs. The same protocol was applied by other authors to evaluate the concentrations of several pesticides and metabolites in human biological fluids [37].

The results obtained to date, although promising, have to be confirmed in larger patient cohort studies in order to be accepted into clinical practice. The purpose of using VCs is to provide non-invasive, early detection of various disease states. GC-MS is mainly used for biomarker discovery; however, it would be difficult to implement in a clinical setting. Hence, for near patient testing, the development of new detection devices or miniaturised MS instruments is required.

### 2.2. Gas Chromatography-Tandem Mass Spectrometry (GC-MS/MS)

**Figure 1:**
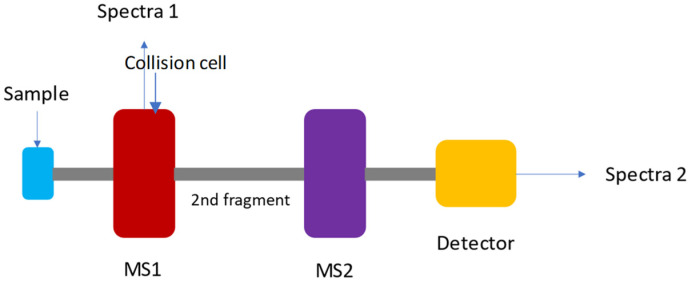
A simple schematic of a tandem mass spectrometry.

In GC-MS/MS, the analytes enter the MS/MS, which consists of two scanning mass spectrometers that are separated by a collision cell. The fragments that are selected in the first analyser are reacted with an inert gas (e.g., He) in the collision cell, resulting in further fragmentation so that the different VCs can be detected (Figure 1). The second fragmentation increases the selectivity and sensitivity of MS/MS over a single quadrupole. Moreover, MS/MS lends itself to the analysis of heavier molecules due to the second fragmentation phase. GC-MS/MS is an excellent instrument for quantitative analysis and is commonly employed for routine targeted analyses. On the contrary, GC-MS/MS has limitations in terms of mass accuracy and resolution, and it should not be the first choice for untargeted analysis.

Wang et al. [38] used GC-MS/MS to study the pharmacokinetics of Longu Rendan pills, a Chinese medicine that is used to prevent heatstroke and motion sickness. Using this technique, the team determined the concentrations of menthol, isoborneol, and borneol. They were able to determine the pharmacokinetics of the drug using the GC-MS/MS technique in an in vivo study [38].

Biagini et al. [39] used GC-MS/MS to detect a range of ketones and aldehydes at a LOD of less than 200 parts per trillion by volume (pptv). This technique was then used to determine carbonyl compounds that had been previously derivatised on-sorbent in the breath of heart failure patients [39]. Aldehyde and ketone concentrations can reflect oxidative stress, which is linked to several diseases such as diabetes, liver diseases, and heart failure.

Despite the benefits of GC-MS/MS, namely the increased sensitivity and specificity and superior ability to identify larger molecular weight molecules versus a single quadrupole, it is not a commonly used technique for biomedical investigations using VCs. However, some examples of studies applying GC-MS/MS for metabolomics with derivatisation of non-volatile compounds can be found [40,41,42,43].

### 2.3. Gas Chromatography-Time of Flight Mass Spectrometry (GC-TOFMS)

In TOFMS, ions are accelerated by an electric field of known strength. The velocity of the ion depends on the mass-to-charge ratio, which in simple terms, means that heavier ions of the same charge will have a lower velocity than lighter molecules with the same charge. The time taken for the ion to reach a detector at a known distance is measured. This time will depend on the velocity of the ion, which corresponds to its mass-to-charge ratio. From this ratio and known experimental parameters, the ion can be identified. The advantages of using TOFMS include increased mass accuracy and mass resolution, greater sensitivity, rapid acquisition, and increased dynamic range when profiling over a broad molecular weight range.

Baranska et al. [44] analysed the breath samples from 1630 participants using GC-TOFMS (170 irritable bowel syndrome (IBS) patients, 153 controls, and 1307 general population). The authors found 16 VCs that could predict IBS in 89.4% of cases [44]. Similarly, Pijls et al. [45] identified a set of 11 volatiles to discriminate patients with chronic liver disease (*n* = 87) from those with compensated cirrhosis (*n* = 34). Over the course of this study, the group claimed a recorded data matrix of 3718 individual compounds from 152 chromatograms [45]. For the validation set, in which seven chronic liver disease and seven compensated cirrhosis were differentiated with a sensitivity of 0.83 and specificity of 0.87 an ROC of 0.90 was determined. While these results are very encouraging, both of these examples lack details about the identification of the VCs.

VC analysis has enormous potential for clinical utility, especially when it is used in conjunction with other tests. Smolinska et al. [46] performed a GC-TOFMS analysis of breath samples from 184 patients and correlated these results with microbiome tests from faecal samples in Crohn’s disease patients. This group demonstrated a link between the metabolites found in the breath and the microbiome found in the stool. This type of analysis with high resolution and accurate instrumentation should help provide a deeper mechanistic understanding of how the VCs can be clinically useful and that it can also provide the pathogenesis of gastrointestinal diseases.

### 2.4. Two-Dimensional Gas Chromatography-Time of Flight Mass Spectrometry (GC × GC-TOFMS)

GC × GC contains two chromatographic ovens that house two separate columns differing in polarity, allowing the better separation of compounds eluting at the same retention time from the first analytical column. The output of this analysis is termed a two-dimensional chromatogram. Coupling together two columns allows the better separation of the compounds versus a standalone GC column. Additionally, GC × GC is also capable of separating chemical isomers. As mentioned, the two columns usually differ in polarity, allowing the sufficient separation of the compounds that are coeluting from a single GC column whilst maintaining high sensitivity and specificity. Combining the separation of GC × GC with the detection of TOFMS provides a powerful tool for metabolomics. However, it is not a tool for less complicated matrices, which can be successfully analysed with a simpler technique. There are several limitations of GC × GC-TOFMS that are dependent on the applied modulator. 

Thermal modulators require the high consumption of liquid nitrogen, which increase per sample cost. In addition, this type of modulator requires an additional method development step in order to optimise the suitable modulation and hot/cold pulse times. 

On the other hand, flow modulators overcome the limitations associated with thermal modulators. However, flow modulators that provide lower sensitivity due to the splitting of the outlet flow can have a lower resolution in the second dimension due to fill/inject design and have a limited sample capacity range, which makes them consequentially easy to overload.

Mellors et al. [47] utilized the GC × GC-TOFMS technique to determine the hypoxia VC signature of *Aspergillus fumigatus* using SPME as a pre-concentration method. They were able to show the difference between early hypoxia (1 h) and late hypoxia (8 h) and early and late normoxia (1 h and 8 h, respectively) [47]. By obtaining comprehensive VC data from this method, this group was able to differentiate the oxygenation state of *Aspergillus fumigatus* using 19 VCs; however, more importantly, these data allowed the group to postulate possible pathways by which the fungus can continue to metabolize under hypoxic conditions involving 2,3-butandione and 3-hydroxy-2-butanone [47]. The idea of proposing metabolic pathways using VC profiles is important, as this could provide crucial information in the pathogenesis of any number of disease states, including infections, cancers, and metabolic disorders.

The same group also conducted similar work by characterising the VC metabolome of *Klebsiella pneumoniae* grown in human blood [48]. In this study, the group used the GC × GC-TOFMS and were able to identify compounds that were not previously reported as being produced by *K. pneumoniae*. This was conducted by comparing the sterile human blood sample to blood from the same participant inoculated with *K. pneumoniae*. The authors reported an increase in VC production in the inoculated blood. By admission, the authors acknowledge that their study is limited by only using the blood from one participant. Moreover, they were able to compare the VCs that were produced by other strains of bacteria grown under the same conditions [48].

### 2.5. Gas Chromatography-Mass Spectrometry/Metal Oxide Sensor (GC-MS/MOS)

Sensors and sensor arrays can have greater sensitivity versus certain MS techniques. This means that sensors can enhance the differentiation of samples into clinically relevant groups. Usually, sensor outputs rely on pattern recognition techniques for data analysis. Moreover, sensors are rarely able to provide qualitative data to support the findings or to infer mechanisms by which a change in the VCs present in a sample may occur. Despite this, the use of sensors could contribute to the development of high throughput, cost-effective, and accurate clinical tests. By combining a sensor and MS detector, it is possible to benchmark the sensor vs. the accepted gold standard, enabling its development. There is also the possibility of gaining qualitative information on the nature of the VCs that the sensor is detecting, which is useful for the future development of point of care devices.

A GC has been reported as being coupled simultaneously to MS and a metal oxide sensor (MOS), increasing the potential of both detectors. A Clarus 500 GC-MS (PerkinElmer, Inc., Gaithersburg, MD, USA) with a single quadrupole detector was used for all of the samples with the GC output split by S-Swafer technology (PerkinElmer, Inc., Gaithersburg, MD, USA), with 50% going to the MS, and 50% going to the MOS sensor. The full assessment of this novel analytical instrument is described elsewhere [49]. When tested to 29 standards across a range of functional groups and masses, 23 of these compounds showed that the MOS sensor had equal or superior sensitivity to MS. Similarly, when tested with the headspace of stool samples and bacterial cultures, the MOS detected more peaks on average than the MS. The enhanced sensitivity of MOS combined with the qualitative data from the MS showed that this instrument has the potential to differentiate samples into clinically relevant groups while also providing metabolic insights [49]. 

To date, only one application of this system has been published [50]. The authors reported that the GC-MS/MOS system could distinguish the extended-spectrum beta-lactamase (ESBL) and non-ESBL producing strains of *Escherichia coli* that are responsible for urinary tract infections. However, despite these promising results, it is difficult to draw definite conclusions about the applicability of GC-MS/MOS in the clinical diagnostics field, and more applications of this system with studies with a bigger sample size are required to confirm this.

## 3. Real-Time Mass Spectrometry Techniques

The use of real-time MS is still a relatively new and emerging field of analytical science. These techniques are usually highly sensitive and are better suited to providing quantitative data. Moreover, the sample analysis time can be reduced to seconds, and in many cases, there is no need to pre-concentrate or prepare samples. However, real-time techniques often lack detailed qualitative data; thus, elucidating metabolic data is difficult. 

Table 2 summarises the advantages and limitations of real-time MS techniques. In general, these techniques can provide accurate quantification at very low levels, making them ideal for the targeted analysis of trace VC. These techniques have numerous applications, such as agri-food, environment, and biomedical applications [28,29]. Since these instruments offer rapid real-time outputs, they are ideally suited for breath analysis, and numerous studies have been performed to reflect this [28]. However, real-time techniques usually require target analytes to achieve quantification, which typically require the use of chromatographic MS techniques to find the target. Moreover, the qualitative information obtained by these instruments is usually only indicative of the compounds that are present and offers no definite identification. Perhaps the biggest challenge faced by real-time MS is the overlap of compounds with the same *m*/*z* value [28].

**Table 2:** Advantages and limitations of the real-time MS techniques.

Instrument	Advantages	Limitations
SIFT-MS	Provides real time quantification of compounds. Simple to operate. Easy to interpret data. Can discriminate isomers. Highly sensitive (pptv levels). Can detect compounds GC-MS cannot, such as amines and thermally unstable compounds. High throughput. No sample pre-processing. Multiple reagent ions.	Quantification relies on having a target analyte with known kinetic parameters. Instruments are often very expensive. Difficult to obtain meaningful qualitative data. Having two quadrupoles under a vacuum can mean more maintenance. Can only measure a limited number of compounds simultaneously.
PTR-MS	No front quadrupole means the cost is reduced versus SIFT-MS and PTR-TOF-MS. High throughput. Can provide quantification within 20% accuracy without compound identification. Provides real-time quantification of known compounds. Can be used in conjunction with GC methods. More sensitive than SIFT-MS. Easy to maintain.	Only use the H_3_O^+^ reagent ion. LOD determination requires instrument calibration with standards. Possible interference from other compounds with same *m*/*z* ratio. Low mass resolution means separation of isomers is not possible. Can only measure a limited number of compounds simultaneously.
PTR-TOFMS	High throughput. Greater mass resolution means isomers can be resolved even in complex matrices. No front-end quadrupole means the instrument is smaller than SIFT-MS. Can rapidly detect the full mass spectrum in one TOF pulse. Data can be mined to find unknown compounds. Greater sensitivity versus SIFT-MS.	Comparable cost to SIFT-MS. Data processing can be complicated and time-consuming. Some reports suggest it has limited sensitivity compared to conventional PTR-MS. Still vulnerable to *m*/*z* overlap in complex matrices.
SESI-MS	Can detect very high mass and very polar compounds. Very sensitive method (up to ppqv). Operates at ambient pressure. Real-time characterisation of VCs.	Concentration calculation not possible. Very expensive instrument. Lower sensitivity for compounds with low proton affinity. In the case of a high number of related volatiles the specificity of the detected VCs can be reduced. External factors can cause false-positive results

### 3.1. Selected Ion Flow Tube Mass Spectrometry (SIFT-MS)

**Figure 2:**
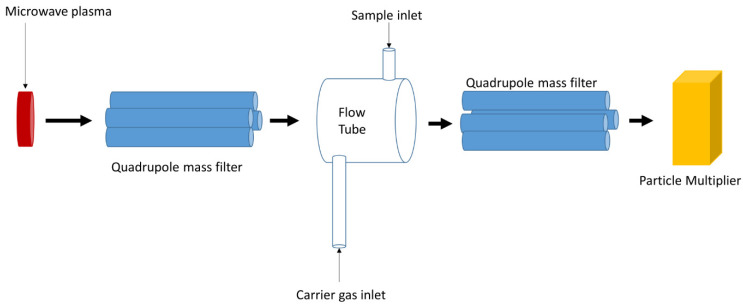
Schematic of a SIFT-MS.

Selected ion flow tube MS (SIFT-MS) provides a real-time quantitative analysis of gases and is capable of detecting a wide variety of analytes in the low part per billion (ppb) range [51]. The manufacturers have reported that the new generation of instruments can achieve part per trillion levels of sensitivity for selected analytes. In contrast to GC-MS, SIFT-MS has no chromatographic phase. The SIFT-MS generates the reagent ions H_3_O^+^, NO^+^, or O_2_^+^ by mixing air and water vapour in a microwave plasma source. The front quadrupole filters these reagents, and the ions to be used are determined by their reactivity with the pre-selected analyte compounds. These reagents then enter the flow tube, where they are reacted with the sample (Figure 2). This soft chemical ionisation process forms ionised molecules that are then focused through a second quadrupole and the MS detector.

Due to the real-time quantitative output and no requirement for a pre-concentration step, SIFT-MS is ideally suited for assessing breath VCs and bacterial headspace analysis. Numerous reports exist on both applications. Spanel and Smith [51] provide a review of SIFT-MS in breath analysis. Breath analysis has been used for the diagnosis of many different disease states such as breast cancer [52], liver disease [53], malignant biliary strictures [54], renal failure [55], chronic kidney disease [56], Crohn’s disease and ulcerative colitis [57], inflammatory bowel disease (IBD) [58,59], and gastro-oesophageal cancer [60].

An interesting study used SIFT-MS to find the difference between gastrointestinal disorders such as inflammatory bowel disease (IBD) (Crohn’s disease (*n* = 24), ulcerative colitis (*n* = 11)), and ileal pouch anal anastomosis (*n* = 30), and non-inflammatory controls (*n* = 53) based on the VCs in breath [61]. The authors were able to differentiate IBD from other gastrointestinal disorders based on the VCs that were in exhaled breath. Relative concentrations of 22 compounds were compared using the SIFT-MS. For Crohn’s disease versus the non-inflammatory controls, 7 out 22 compounds were significantly different (2-propanol, acrylonitrile, carbon disulphide, dimethyl disulphide, ethanol, isoprene, and triethylamine). Importantly, they showed that Crohn’s disease and ulcerative colitis could not be distinguished from one another and that the VC profile was not altered by the inflammation in ileal pouch anal anastomosis [61].

In 2017, Demirjian et al. [55] used SIFT-MS breath analysis to identify a breath-print in patients with end-stage renal disease. This work showed that they were able to distinguish end-stage renal disease patients from healthy controls with a C statistic of 0.99. While this is a very encouraging result, it should be noted that only 86 patients with renal disease were analysed and that only 25 healthy volunteers served as controls. While it would be beneficial to identify the different grades of renal disease from the early stages onward, that process would require numerous patients with a range of clinical stages of illness, which is time-consuming and very costly.

SIFT-MS has been used for the online breath monitoring of acetone, isoprene, and acetic acid while undergoing surgery, with an operation period ranging in time from 20 min to 60 min [62]. This work showed that with the exception of isoprene, the compounds remained relatively stable. Isoprene, however, doubled in concentration in most cases over the course of the surgery [62]. This work showed that SIFT-MS is not only useful for single sample analysis but also for continuous monitoring over time.

Sovova et al. [63] used the continuous monitoring of bacteria from three species (*Serratia rubidea*, *Serritia marcescens*, and *E. coli*), and several VCs including ammonia, ethanol, acetaldehyde, propanol, acetone, and acetic acid were quantitatively analysed for a continuous 24 h period. The authors were able to determine that the headspace composition could determine the bacterial species. For instance, the presence of propanol was indicative of an *E. coli* population. This work also demonstrates that the growth and death of the bacterial cultures could be monitored without interfering with the sample [63]. Similarly, air changes in the atmospheric compounds can also be monitored over time, as in the study of Prince et al., who measured the concentration of toluene, 1,3-butadiene, benzene, ethanol, and ethene over five days [64]. This group was able to record 1,3-butadiene at a level of 9 pptv with ±4 pptv precision and demonstrated the capabilities of SIFT-MS for the online real-time monitoring of VCs [64].

SIFT-MS allows not only the calculation of the concentration of selected compounds but also allows users to perform a full scan that can show the *m*/*z* peaks present in the sample, which can give some indication of the compounds that are present. In full scan mode, the SIFT-MS detects the mass to charge ratio (*m*/*z*) for VCs across a selected mass range (e.g., 30–200). The measurement limit for each mass unit can be set to a function of time (milliseconds) or ion counts; optionally, it can be set to whichever is reached first. In full scan mode, the SIFT-MS scans H_3_O^+^ and O_2_^+^ reagent ions sequentially over the course of the sample run; this parameter cannot be altered on some instruments.

In this mode, the SIFT-MS has limited qualitative and quantitative ability and instead provides an output of *m*/*z* versus ion count, providing a distinct profile for each sample. As the SIFT-MS uses a soft chemical ionisation, knowing the *m*/*z* of the ions present can provide clues towards the possible compounds in the sample.

However, while searching the literature for this paper, we were unable to find any examples in which full scan mode had been reported.

### 3.2. Proton Transfer Reaction-Mass Spectrometry (PTR-MS)

**Figure 3:**
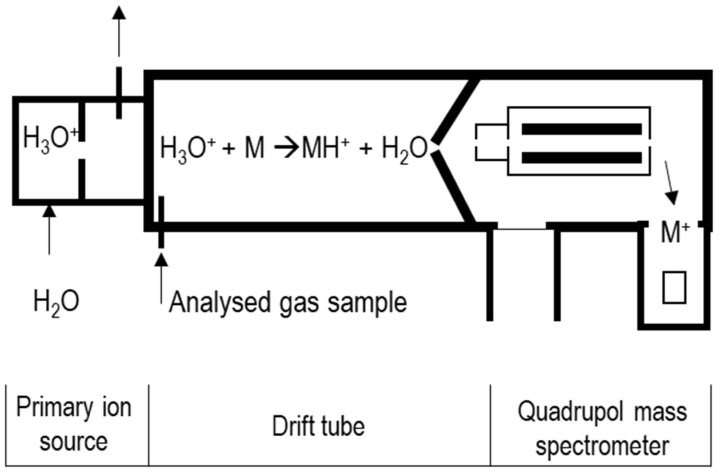
A simple schematic of a PTR-MS.

Proton transfer reaction mass spectrometry (PTR-MS) is a similar technique to SIFT-MS, as it is a real-time soft chemical ionisation of compounds that results in less compound fragmentation than traditional quadrupole MS [65]. Similar to SIFT-MS, in most cases, there is no need for sample preparation. The generation of H_3_O^+^ is similar to that of the SIFT-MS; however, in a PTR-MS system, there is no filtering quadrupole and no flow tube (Figure 3). Instead, the ions and analyte VCs are mixed in a drift cell (or drift tube) with controlled pressure, temperature, and magnetic field; thus, there is no need for a carrier gas, which results in a claimed sensitivity that is two orders of magnitude greater than SIFT-MS [65]. The ion focusing interface moves the ions from the drift cell into the mass analyser; this process is essential, as the drift cell has a pressure of approximately 2 mbar, while the analyser operates at 1 × 10^−6^ mbar. In this interface, the ion beam moves between several stages of decreasing pressure in order to reconcile the pressure difference (Figure 3).

Winkler et al. [66] demonstrated how this real-time technique, with its excellent sensitivity (pptv), could be used to elucidate the metabolism of the VCs in the body. In their study, isotope-labelled ethanol was ingested, and the breath was monitored using a PTR-MS instrument. After 10 min, metabolic products from the breakdown of the labelled ethanol could be seen (deuterated acetone and isoprene).

In another study, del Rio et al. [67] compared the breath of cirrhosis patients before and after liver transplant using PTR-MS. Overall, the group showed that limonene, methanol, 2-pentanone, 2-butanone, and carbon disulphide decreased following a successful transplant. While limonene had the best individual diagnostic ability (ROC 0.91), this was enhanced when combined with methanol and 2-pentanone (ROC 0.95). This study demonstrated that while an individual VC may serve well as a biomarker, the likelihood is that a combination of markers will allow for greater discriminatory capabilities.

More recently, Zou et al. [68] used an ultrasonic nebulisation technique to analyse urine using PTR-MS. The authors analysed methanol, acetaldehyde, and acetone from healthy human urine, which yielded a recovery of 88.89% and 94.54% based on the concentrations that were obtained from the previous work of the group. This method requires only 0.66 mL of urine, making it ideal for patients struggling to provide bigger sample volume, as some patients who are under stress, dehydration, or simply feeling unwell can fail to provide significant volumes of urine [68]. Moreover, this method yields LODs in the low μg/L (tested with standards of methanol LOD 4.47 µg/L, acetaldehyde LOD 1.98 µg/L, and acetone LOD 3.47 µg/L) and can provide a full scan result in 34 s.

### 3.3. Proton Transfer Reaction-Time of Flight Mass Spectrometry (PTR-TOFMS)

PTR can also be used in combination with a TOFMS detector instead of a traditional single quadrupole MS. It allows all the benefits of the PTR system to be retained and combines them with the greater resolution and mass range of the TOFMS.

In a study using PTR-TOFMS to differentiate coeliac disease patients from healthy controls, Aprea et al. [69] were unable to find any significant differences between the two groups. However, the coeliac disease patients were all following a gluten-free diet and were asymptomatic; thus, the inflammatory state in the gut may have been alleviated, resulting in the lack of differences between the groups.

A more recent study also used PTR-TOF-MS to investigate the dietary impact on the breath volatilome. Kistler et al. [70] found that diet-induced obesity altered the VC profile in mice. The high-fat diet resulted in the elevated concentration of keto-bodies in the breath, which are considered to be markers of lipid peroxidation. Moreover, the concentration of (methylthio)-methanethiol was reduced, indicating a link between a high-fat diet, metabolic changes, and reproduction [70]. The authors suggested that breath can be used to not only diagnose potential metabolic changes early but to also monitor the progression and regression of these changes in obesity cases.

The idea of an attempt to understand the metabolic processes by determining how and why metabolites are generated in the body was further explored by Obermeier et al. [71] in a study with patients with different stages of chronic kidney disease (CKD). Individuals with stage 1 CKD had elevated levels of ammonia, while patients in the later stages (2–4) had higher levels of isoprene, pentanal, and heptanal. Methylamine was lower in patients with CKD compared to the controls (either patient post-transplant or with acute infections) [71]. The promising results of this study showed the potential of the early detection of metabolic changes associated with CKD. The idea of detecting a disease such as CKD in the early stages is often crucial for improved recovery outcomes.

### 3.4. Secondary Electrospray Ionisation-Mass Spectrometry (SESI-MS)

Another technique applied in the VC analysis field is secondary electrospray ionisation (SESI-MS), a variant of electrospray ionisation. SESI-MS consists of an electrospray plume of pure acidified solvent that interacts with neutral vapours. Vapour molecules become ionised at atmospheric pressure when the charge is transferred from the ions that are formed in the electrospray to the molecules. The main advantage of SESI-MS is that it can detect compounds of low volatility with molecular masses as high as 700 Da that are present in the sample at very low concentrations. SESI-MS allows for the analysis of samples in real-time and non-invasively and has been found to be the most sensitive for polar compounds reaching the sub part per quadrillion per volume (ppqv) [29]. 

Therefore, the number of studies using SESI-MS for breath gas analysis, biomarker discovery, and in vivo pharmacokinetic studies has increased in recent years. On the other hand, a high number of related volatiles can reduce the diagnostic specificity of detected VCs, and external factors can cause false-positive results, and the ESI-MS operators should limit the use of fragrant personal care items such as perfumes, mouthwash, lotions, gum, cigarettes, etc., before using the instrument, as fragrance can affect the instrument response [29,72]. However, the main limitation of SESI-MS is the high cost of the instrument, which limits its availability.

SESI-MS has been repeatedly reported as a robust tool for cancer identification based on breath analysis [73,74,75]. An interesting study was described by Bruderer et al. [76], who compared the potential of SESI-MS and PTR-MS for VCs analysis in the breath. SESI-MS detected the highest number of compounds in the high mass region (*m*/*z* = 150–250), while PTR-MS was more sensitive in the low mass region (*m*/*z* = 50–150). PTR-MS allowed for the detection of 491 peaks, whilst SESI-MS detected almost double that number (828). Interestingly, only 133 peaks were common for both techniques, and 797 and 374 unique features were detected for SESI and PTR, respectively [76].

## 4. Discussion

This review highlights multiple examples of MS applications for VC analysis in the biomedical sector. Table 3 summarises the advantages and disadvantages of hyphenated and real-time MS for VCs analysis. While the hyphenated MS-based techniques require more sample preparation and generally have longer analysis time per sample, the metabolic insights that they can deliver with their qualitative data make the cost to benefit worthwhile. The qualitative data and the identification of VCs are crucial to understanding the metabolic changes during disease states and thus to understanding how VCs can be used in the clinical forum. The quality of data the that can be collected from these instruments makes the shortcomings, such as the set-up cost, method development, and in most cases, long sample analysis time, worthwhile. On the other hand, real-time techniques are generally simple to operate, and this can be undertaken by semi-skilled operators. While hyphenated techniques can also be easy to operate, they require significantly more method development and more complex spectral analysis, which does require more expertise. Moreover, due to the real-time nature, the time of analysis per sample is very short, making them ideal for high throughput clinical laboratories. It is also possible to bring the patient to the instrument for breath analysis.

In order to obtain quantitative data from, e.g., SIFT-MS, the analyte compounds must be selected, and any other compounds present will not be monitored. Thus, for the SIFT-MS to be clinically useful, the biomarkers must be found, identified, and validated, which can only be achieved using chromatographic MS techniques.

However, the data collected from real-time techniques often require algorithms or pattern recognition methods for the direct differentiation of disease, though this also applies to chromatographic methods when comparing whole chromatograms, i.e., not undertaking an exhaustive qualitative analysis of the chromatographic peaks. For example, there are many metabolomic-based software programmes [77,78] that have been developed to enable the analysis of groups of total ion chromatograms, i.e., diseased vs. non-diseased. The software usually bases this analysis on differences in specific ion intensity signals, which circumnavigates some issues of peak co-elution and subjective library matching, which can exist when trying to extract qualitative data.

The large library databases for GC-MS are both a positive and potential drawback when undertaking non-targeted biomarker screening [79]. For example, even though they are extensive, they only contain a fraction of the possible VCs [21]. Additionally, the library will match to a certain compound regardless of the probability of the match being correct, and this tends to become lower when the molecular weight, branching, etc. [80], increase. Therefore, the qualitative data from the library coupled with retention indices need to be carefully analysed or ideally confirmed with standards to confirm metabolite identification. In contrast, many techniques such as the GC-TOFMS, GC × GC and MS/MS techniques were limited in the past when compared to quadrupole GC-MS by the mass spectral libraries that were available although recent software developments [81] have enabled improvements [82]. As an example, it was possible to detect 1000 s of chromatographic peaks when analysing breath samples by GC × GC-TOFMS [83], but naming even a fraction of these proved difficult. However, it did highlight the issue with one dimensional GC-MS techniques and the numerous co-elutions when analysing complex clinical samples. Additionally, it should be noted that many TOF-based MS instruments offer accurate mass, which helps in determining a likely chemical formula when compared to quadrupole instruments [84].

Likewise direct-MS techniques tend to have very limited libraries vs. chromatographic MS techniques, and although these are constantly being added to by instrument manufacturers and experienced users (requiring multi-point calibrations with standards), it limits their usefulness for biomarker discovery in clinical studies [85]. However, when used in conjunction with hyphenated MS-based techniques, they can provide quantitative real time data for previously validated markers. One of their true benefits for clinical analysis is their ability to analyse these clinical markers over extended time periods, e.g., dynamic monitoring of patient breath/bacterial growth curves of isolates [85]. To attempt to undertake the same studies using hyphenated MS systems would require the construction of calibration graphs and multiple point measurements of VOCs, which even then would not give a clear physiological/clinical picture.

With the further development of mass spectral libraries, we envisage GC × GC-TOFMS superseding GC-MS as the gold standard for the qualitative analysis of clinical samples. This is due to the superior mass resolution, sensitivity, and superior chromatographic separation of the complex matrices that are afforded by GC × GC-TO-MS versus GC-MS. It is also worth noting that PTR-MS instruments have been developed for use with additional reagent ions, which extends the range of compounds that can be analysed in line with SIFT-MS [86,87]. As PTR-MS is coupled with the superior TOF-MS analyser versus the quadrupole on the SIFT-MS, it gives it some potential advantages. PTR-MS also has the potential to occupy a smaller footprint due to a vacuum system that is reduced in size and that does not require a carrier gas. Further reductions in size for direct-MS instruments could mean that they could be deployed for near patient testing. SESI-MS also has the potential to occupy a smaller footprint and to be deployed for near patient testing. In addition, SESI coupled with high resolution MS (HRMS) has the ability to undertake the qualitative analysis of unknown clinical samples by deploying MS/MS techniques [88]. It can also be used directly with an extended range of samples, potentially making it a versatile technique for future clinical analyses. There is also the development of new front-end methods such as coupling TD with SIFT-MS and SESI with TOF-MS [89,90]. There is evidence that suggests the competition between certain analytical methods drives innovation and product development, especially for clinical applications.

**Table 3:** A summary of the main advantages and disadvantages of chromatographic and real-time MS techniques.

Instrument	Advantages	Disadvantages
Hyphenated MS	Unparalleled qualitative analysis.	Requires more sample preparation and method development.
	Highly sensitive.	Long analysis time.
	Capable of analysing complex matrices with unknown compounds.	Requires skilled operators to develop methods and get the best out of the data. Can only analyse compounds that are thermally stable and volatile.
Real-time MS	Capable of providing a simple method to quantify compounds.	Quantification requires a target analyte.
	Simple to operate. Can analyse and quantify thermally unstable compounds.	Full-spectrum data requires skilled data analysis for pattern recognition. Requires compounds that are volatile at lower temperatures.
	Very short sample analysis time allows for high throughput.	Lack of accurate qualitative data makes complex matrices difficult to analyse.

An important aspect of biomarker discovery for diagnosing disease is understanding what constitutes a healthy profile in humans. Although there is a qualitative understanding of approx. 2000 compounds that have been identified, only a small fraction of these have been validated by standards [13]. Furthermore, there is no understanding of the concentration ranges of the vast majority of these compounds. Until this is established, it will limit the clinical utility of volatile analysis. If the number of papers dealing with clinical analysis utilising VCs is contrasted with the low numbers of FDA-approved tests [91], then based on VC analysis, it can be seen that this field remains in the early stages of development. Current FDA-approved breath tests with clinical utility include C4–C20 alkanes for heart transplant rejection, ethanol on breath, nitric oxide for airway inflammation, carbon monoxide for smoking status, and methane and hydrogen for sugar malabsorption [91]. Some of these VCs, such as ethanol, are easily quantified using direct-MS techniques, and their levels have been measured in large cohorts of the general population. Other compounds such as nitric oxide and hydrogen are difficult to measure via SIFT-MS and PTR-MS, whereas good electrochemical sensor technology and analytical methods exist for their accurate quantification [92]. The C4–C20 alkanes were identified using GC-MS [93] and could be directly quantified in breath by direct MS [94].

Therefore, one of the main conclusions is that clinical utility will be enhanced when fundamental studies are undertaken to identify the full range of VCs that are associated with healthy humans (using hyphenated MS techniques) and their concentration ranges in healthy humans (using direct MS techniques). Fundamental work to understand the metabolic pathways that liberate the VCs, using isotope-labelled substrates for example, should be undertaken [95]. In addition, fundamental studies to understand the effect of the microbiome [96] as well as environmental exposure [97] will all help underpin the clinical utility. These should run in parallel to the studies that are aimed at identifying markers of disease, which are currently more prevalent.

## 5. Conclusions

In summary, each of the listed methods has its strengths and limitations and can be chosen based on the specific clinical application. Coupling chromatography-based and real-time techniques can increase the analytical potential of the obtained system. In the field of analytical chemistry, new developments are proposed each year. Several successful combinations of PTR-MS with GC-MS were reported, allowing a clear interpretation of the complex PTR-MS spectra [98]. However, has not yet been used for diagnostic purposes. There was also the combination of GC × GC-TOF-MS and SIFT-MS for breath analysis [99]. Moreover, fast GC techniques are being developed that allow sufficiently short run times to be achieved that are in the range minutes to seconds, thus achieving close to real-time analysis [100].

Confirmed biomarkers, identified using GC-based techniques and that can be efficiently quantified using real-time MS techniques, will potentially allow robust biomarker discovery, which would enable the targeted fabrication of selective sensors that could be deployed for point of care testing. Alternatively, the further reduction of the current instrument footprints, particularly for direct-MS techniques may enable their direct use for near-patient testing.

## Abbreviations

CKD	chronic kidney disease
EI	electron impact ionization
ESBL	extended-spectrum beta-lactamase
FDA	U.S. Food and Drug Administration
GC-MS	gas chromatography-mass spectrometry
GC-MS/MOS	gas chromatography-mass spectrometry/metal oxide sensor
GC-MS/MS	gas chromatography-tandem mass spectrometry
GC-TOFMS	gas chromatography-time of flight mass spectrometry
GC × GC-TOFMS	two-dimensional gas chromatograph-time of flight mass spectrometry
HS	headspace
HRMS	high resolution mass spectrometry
IBD	inflammatory bowel disease
IBS	irritable bowel syndrome
LC	liquid chromatography
LLE	liquid-liquid extraction
LOD	limit of detection
LPME	liquid phase microextraction
MALDI	matrix-assisted laser desorption/ionization
MOS	metal oxide sensor
MS	mass spectrometry
*m*/*z*	mass to charge ratio
NIST	The National Institute of Standards and Technology
ppb	part per billion
ppqv	part per quadrillion per volume
pptv	parts per trillion by volume
PTR-MS	proton transfer reaction-mass spectrometry
PTR-TOFMS	Proton transfer reaction-time of flight mass spectrometry
ROC	receiver operating characteristic
SBSE	stir bar sorptive extraction
SDME	single-drop microextraction SPDE: solid-phase dynamic extraction
SESI-MS	secondary electrospray ionization—mass spectrometry
SIFT-MS	selected ion flow tube-mass spectrometry
SPME	solid-phase microextraction
TD	thermal desorption
VASE	vacuum-assisted sorbent extraction
VCs	volatile compounds
